# An Evaluation of Duplicate Adverse Event Reports Characteristics in the Food and Drug Administration Adverse Event Reporting System

**DOI:** 10.1007/s40264-025-01560-7

**Published:** 2025-06-04

**Authors:** Scott Janiczak, Sarah Tanveer, Karen Tom, Rongmei Zhang, Yong Ma, Lisa Wolf, Monica A. Muñoz

**Affiliations:** 1 Office of Surveillance and Epidemiology (OSE), Center for Drug Evaluation and Research (CDER), US Food and Drug Administration, Silver Spring, MD USA; 2 Office of Strategic Partnerships and Technology Innovation, Center for Devices and Radiological Health, US Food and Drug Administration, Silver Spring, MD USA; 3 Office of Biostatistics, US Food and Drug Administration, Silver Spring, MD USA

## Abstract

**Introduction:**

The Food and Drug Administration (FDA) Adverse Event Reporting System (FAERS) receives duplicate reports of adverse events associated with drug and therapeutic biological products. Duplicate reports, defined as multiple reports of the same adverse event(s) related to the administration of the same marketed product(s) to the same individual patient at a particular point in time, may be received in FAERS for many reasons. The presence of duplicate reports can negatively impact public health surveillance efforts by impeding both safety signal identification and signal evaluation.

**Objectives:**

To characterize the features and contributing factors associated with duplicate reports in FAERS.

**Methods:**

We manually assessed a convenience sample of individual case safety reports (ICSRs) for duplication, resulting in two data sets: one consisting of non-duplicate reports and one with duplicate reports. We then compared key features of these two datasets, including both structured and unstructured data fields. Key comparison features included: report and reporter type, country of report origin, data source for report, and outcome. In addition, we evaluated information similarity of reports for seven data elements (e.g., age, sex, suspect products) within sets of duplicates using both structured and unstructured fields. We used pairwise sentence bidirectional encoder representations from transformers (SBERT) cosine similarity scores to examine free-text narrative similarity.

**Results:**

Among the 2297 reports in the sample, 901 (39%) were classified as duplicates, consisting of 237 unique duplicate sets. Compared to non-duplicate reports, duplicates were more likely to be foreign reports (82% versus 37%), reported by healthcare professionals (89% versus 68%), mention other regulatory authority databases (42% versus 11%), describe published case reports (34% versus 11%), or have a serious outcome (97% versus 83%) (*p* < 0.0001). Within sets of duplicates (*n* = 237), coded information was frequently different, with only 16% (*n* = 39) having concordance of all 7 data elements. The narrative was highly similar among most sets of duplicates; we found that the median similarity score for the duplicate pairs was 0.87 compared to 0.48 for non-duplicate pairs.

**Conclusions:**

We observed differences in the attributes of and potential contributors to duplicate reports in FAERS that may inform duplicate prevention, detection, and management strategies. However, further studies are needed to better understand the implications of these findings and how potential regulatory changes and technological advances can be leveraged to further address duplicate reporting in adverse event reporting systems.

**Supplementary Information:**

The online version contains supplementary material available at 10.1007/s40264-025-01560-7.

## Key Points

**Table:** 

This study provides insights into the features and contributing factors for duplicate individual case safety reports submitted to the FDA Adverse Event Reporting System.
The evaluated duplicate reports were often derived from other regulatory authorities’ databases and were more likely to be foreign reports, reported by healthcare professionals, or to be case reports published in the literature.
Our study revealed significant variability within duplicate report sets, with limited concordance across key data fields essential for case identification and management.

## Introduction

The Food and Drug Administration (FDA) Adverse Event Reporting System (FAERS) receives duplicate individual case safety reports (ICSRs) (henceforth referred to as reports) of adverse events associated with drug and therapeutic biological products; however, the true extent is unknown [1]. Duplicate reports, defined as multiple reports of the same adverse event(s) related to the administration of the same marketed product(s) to the same individual patient at a particular point in time, may be received in FAERS for many reasons. For example, the same adverse event may be submitted by different reporters (e.g., consumer and their healthcare professional) directly to FDA or the same report may be submitted directly to FDA and to a manufacturer, who must submit the report to FDA according to regulatory requirements; or follow-up to a previously submitted report may be inadvertently coded as a new report with a new case number as opposed to linking it to the original report. In addition, multiple manufacturers of the same product or different suspect products may play a role in duplicate reporting when they submit the same identified case report.

The presence of duplicate reports in adverse event reporting systems can negatively impact public health surveillance efforts by impeding both safety signal identification and signal evaluation [2, 3]. Duplicate reports can affect safety signal detection in that they may result in both false positive signals, and more insidiously, false negatives (as the number of background cases may be inflated due to duplicates) [4, 5]. Duplicates must also be accounted for when developing a case series to ensure the accurate representation of data when evaluating a signal [6]. Prior research found that of a cohort of pharmacovigilance (PV) reviews containing FAERS case series, half of the reviews noted at least 10% of reports were duplicates and about a quarter of reviews identified more than 25% of reports as duplicates [7]. Significant effort by marketing authorization holders (MAHs), regulators, and other PV organizations is spent on de-duplicating reports, which creates additional administrative burden, as review of key data fields, in addition to manually examining case narratives, can be resource intensive.

Although researchers have endeavored to quantify the extent of duplicate reports in adverse event reporting systems and develop tools to facilitate accurate identification, there is currently no universal solution. External research has been hindered by limitations in the availability of data fields in publicly accessible datasets. However, even with complete and unredacted data, the task of detecting and confirming duplicate reports remains challenging. Slight variations in the information reported, coupled with frequently missing data, often leave even the most experienced assessors with a judgment call [8–11]. Better understanding of the characteristics of duplicate reports in the FAERS database may help improve the analytical and scientific approaches used to de-duplicate reports, ultimately improving postmarketing safety surveillance. This study aims to characterize the features and contributing factors associated with duplicate reports within a relatively large manually curated dataset of FAERS reports.

## Methods

### FAERS Data

The dataset included 2297 reports submitted to FAERS that were derived from 12 different FDA PV reviews assessing different drug or drug class–event pairs that were completed in 2020. Reviews were selected to encompass various therapeutic areas, manufacturers, and drug–event combinations. PV reviews are developed in response to a signal identified in the postmarketing period using data such as FAERS in accordance with the Center for Drug Evaluation and Research’s policies and procedures for Newly Identified Safety Signals (NISS) [12]. The reports sourced from each of the aforementioned PV reviews were assessed by two of four total reviewers, who used expert opinion to adjudicate reports as duplicates (*n* = 901; 237 duplicate sets) or non-duplicates (*n* = 1396). A separate adjudicator checked disagreements between the two reviewers and made a final decision on the groupings.

### Descriptive Analyses

We collected the following characteristics from both the duplicate and non-duplicate report cohorts: type of report (i.e., direct, expedited 15-day reports, non-expedited reports), country of report origin, data source for a report, reporter type [i.e., healthcare professional (HCP), consumer/non-HCP], and outcome as noted by the reporter (i.e., serious, non-serious). Data source for a report was determined during manual review and was defined as the data source from where the report information originated and was categorized as: regulatory authority reports, medical literature, clinical study, industry sponsored program (e.g., patient support program) and/or specialty pharmacy, social media, or other data source. We used the initial or first report, even if follow-up was obtained from a different source or reporter, to classify the report source and reporter type. Serious adverse drug experiences (also referred to as serious outcomes) were based on the regulatory definition in Title 21 of the Code of Federal Regulations, 314.80, and include any of the following outcomes: death, life-threatening event, hospitalization (initial or prolonged), disability, congenital anomaly, and other serious important medical events [13]. The report characteristics examined were informed by prior research on duplicates and our collective experience in PV [2, 4, 5].

We performed additional analyses of the duplicate reports. We analyzed the size of each duplicate set and the percentage of duplicate reports within each of the 12 PV reviews. To explore the consistency of information among the duplicate reports in the set, we compared certain data elements that are available in key structured data fields and the narrative similarity between reports. The data elements included: age, sex, country of report origin, suspect products, concomitant products, outcome as noted by the reporter (i.e., serious versus non-serious outcome), and adverse events coded using Medical Dictionary for Regulatory Activities (MedDRA^®^) Preferred Terms (PTs). MedDRA^®^ is the international medical terminology developed by the International Council for Harmonisation of Technical Requirements for Registration of Pharmaceuticals for Human Use, and adverse events within the reports may be coded with MedDRA^®^ PTs by the manufacturer or FDA depending on the type of report (e.g., expedited and non-expedited reports are coded by the manufacturer, direct reports are coded by FDA). We abstracted this information from key structured data fields; if data within the fields was missing for age or sex (e.g., null, unknown, not reported), we manually reviewed unstructured case narratives for the availability of this information. If the data element, such as products or PTs, were reported consistently across each report within a given duplicate set, we classified the set as being concordant. If they were not reported consistently, we classified the set as being discordant. In addition, we compared concordance among data sources and reporter type and further examined the types of serious outcomes among the reports in a duplicate set; however, we did not include these elements in the evaluation of overall concordance. To examine narrative similarity, we employed natural language processing (NLP) techniques to assess how similar narrative text was between reports within a duplicate set.

Lastly, we tabulated two additional characteristics, the total number of available/marketed products and corresponding application types [New Drug Application (NDA), Abbreviated New Drug Application (ANDA), Biologics License Application (BLA)] for the products of interest, and examined their impact on duplicate reporting as these factors have previously been hypothesized as contributors [14]. For this analysis, we included the number of products approved/marketed during the FAERS search time period utilized in the respective PV review for the drug/drug class or biologic of interest.

### Statistical Analyses

Statistically significant differences between duplicate and non-duplicate reports characteristics were tested using a two-sided Chi-squared test for categorical variables. We used a threshold of a *p*-value less than 0.05 to identify possible differences. Statistical analyses were performed using SAS^®^ 9.4 (SAS Institute, Cary NC).

**Fig. 1:**
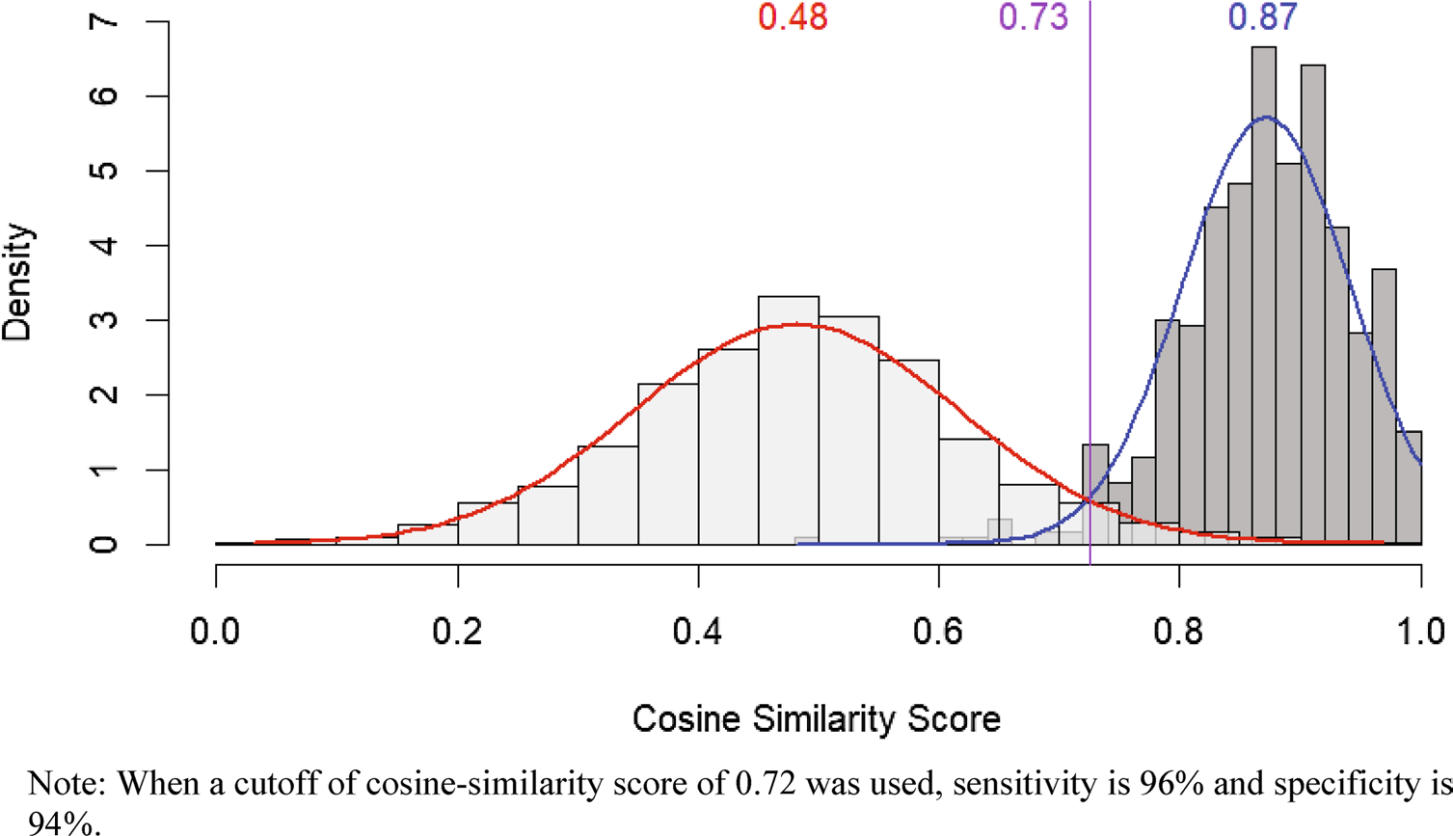
Distribution of cosine similarity analysis of narrative text

For the narrative similarity analysis, we used Sentence-BERT (SBERT), a variant of bidirectional encoder representations from transformers (BERT), to generate sentence embeddings for all report narratives (i.e., our dataset did not include any reports with a missing narrative) [15]. The all-MPNet-base-v2 model from the Sentence-Transformers library was employed with default parameters. Cosine similarity was then calculated between embeddings of duplicate report pairs. Cosine similarity scores between pairs of randomly selected reports from the 1396 non-duplicate reports group were also calculated as a reference measure for comparison. Normal distribution was used to approximate the density distribution of the cosine similarity scores for presentation in Fig. 1. We used a cutoff of the cosine-similarity score at 0.72, which captures 96% of all duplicates (sensitivity 96%) but will also mistakenly capture 6% of non-duplicates (specificity 94%). In addition to the aforementioned primary analysis, we also conducted a post hoc analysis to examine narrative similarity between duplicate pairs and non-duplicate pairs within a single PV review. We used a cutoff of the cosine similarity score at 0.76, which captures 92% of all duplicates (sensitivity 92%) but will also mistakenly capture 30% of non-duplicates (specificity 70%). These statistical analyses were performed using Python (Version 3.9.4).

## Results

We assessed 2297 FAERS reports, of which 901 (39%) were duplicates consisting of 237 unique duplicate sets. On average, there were three reports per duplicate set, and a median of two reports per duplicate set. The largest set contained 21 duplicate reports.

The overall descriptive characteristics of the duplicate and non-duplicate reports groups are presented in Table 1. Compared to non-duplicate reports, duplicates were more likely to be foreign reports (82% versus 37%), be reported by healthcare professionals (89% versus 68%), mention other regulatory authority databases (42% versus 11%), describe published case reports (34% versus 11%), or have a serious outcome (97% versus 83%) (*p* < 0.0001). Among duplicate reports whose primary source of information was deemed to be from a regulatory authority, most mentioned foreign regulatory databases, such as the European Medicines Agency (EMA) EudraVigilance, Canada Vigilance Adverse Reaction Online database, or regional PV centers. Overall, non-duplicate reports were predominately submitted to FAERS from pharmaceutical manufacturers or directly to the agency without a clear data source (60%, *n* = 835).

**Table 1:** Characteristics of duplicate and non-duplicate reports (*N* = 2297)

Characteristic (*n*, %)	Duplicate reports (*n* = 901)	Non-duplicate reports (*n* = 1396)	*p*-value
*Type of report*^a^			< 0.0001
Direct	21 (2)	98 (7)	
Expedited (15-day)	832 (92)	1004 (72)	
Non-expedited	48 (5)	294 (21)	
*Country of report origin*			< 0.0001
USA	162 (18)	874 (63)	
Foreign	739 (82)	522 (37)	
*Reporter type*			< 0.0001
Healthcare professional	802 (89)	943 (68)	
Consumer/non-healthcare professional	99 (11)	453 (32)	
*Data source for report*			< 0.0001
Regulatory authority reports	380 (42)	152 (11)	
Medical literature	304 (34)	157 (11)	
Clinical study	39 (4)	97 (7)	
Industry sponsored programs^b^	15 (2)	132 (9)	
Social media	–	23 (2)	
Other data source^c^	162 (18)	835 (60)	
*Serious outcome reported*^d^			< 0.0001
Yes	873 (97)	1159 (83)	

*FAERS* Food and Drug Administration Adverse Event Reporting System, *USA* United States of America

^a^Direct reports are voluntarily submitted to FDA by consumers and healthcare professionals through MedWatch. Expedited reports and non-expedited reports are reported by manufacturers in accordance with regulatory requirements

^b^Industry sponsored programs include patient support programs and/or specialty pharmacy

^c^Other data source include reports submitted to FAERS from pharmaceutical manufacturers or directly to the agency that did not have an identifiable primary report source

^d^A serious outcome is defined as per 21 CFR 314.80 [13]. An ICSR may be associated with more than one outcome

Information similarity among duplicate reports in a set varied by data element (Table 2). Concordance among reports in a set was highest for country of report origin (97%,* n* = 229), followed by sex (94%,* n* = 223), and age (89%,* n* = 210). Of note, age and/or sex was abstracted from the case narrative for 120 reports that did not contain this information in structured fields; after correcting these inputs, concordance for age and sex increased by 14% and 12%, respectively, and 3 additional duplicate sets became concordant (i.e., composite score improved from 36 to 39 sets). Overall, only 16% (*n* = 39) of duplicate sets had concordance of all seven data elements.

**Table 2:** Concordance in duplicate report sets (*N* = 237)

Data element	Concordant sets *n* (%)
Preferred terms	69 (29%)
Suspect products	166 (70%)
Concomitant products	147 (62%)
Age^a^	210 (89%)
Sex^b^	223 (94%)
Country of report origin	229 (97%)
Outcome as noted by the reporter^c^	143 (60%)
*Composite score*^*d*^	*39 (16%)*

^a^Case narratives were used to identify patient age in 72 reports missing age in the structured field; after correcting these inputs, 34 additional sets became concordant for age

^b^Case narratives were used to identify patient sex in 48 reports missing sex in the structured field; after correcting these inputs, 28 additional sets became concordant for sex

^c^Serious versus non-serious

^d^Composite score represents complete agreement between all data elements for reports contained within a given duplicate set

Further analysis of duplicate sets showed that 80% (189 sets) and 75% (177 sets) had concordance among reporter and data sources, respectively. Although evaluating regulatory outcome as a binary outcome (i.e., serious versus non-serious) showed high concordance, discordance increased if the individual serious outcomes were compared across reports in a duplicate set. Among the 222 duplicate sets coded as serious, 94 sets had a mismatch in the specific types of outcome reported; for example, one report in the set had a serious outcome of “hospitalization” whereas another report in the set had the serious outcomes of “hospitalization and life-threatening.”

Our analysis examining similarity of narrative text between reports found that the median cosine similarity score for the duplicate pairs was 0.87 as compared with a median cosine similarity score for the non-duplicate pairs of 0.48 (Fig. 1). For reference, we have provided the redacted narratives for two sets of duplicates with a cosine similarity score of 0.93 and 0.78, respectively (see Online Resource, eTable 1). In our post hoc analysis examining within-review pairs, we found that the median cosine similarity score for the duplicate pairs was 0.87 as compared with a median cosine similarity score for the non-duplicate pairs of 0.67 (see Online Resource, eFig. 1).

Lastly, we observed a complex relationship between the total number of available/marketed products and the proportion of duplicate reports across all reviews (Table 3). While reviews with the highest proportions of duplicates (Reviews 12, 3, and 2) generally had a larger number of products available, this pattern was not consistent. Notably, Review 1, with only 1 available product had a higher proportion of duplicates (31%) than Reviews 5 and 6, which each had 13 products but lower duplicate proportions (15% and 28%, respectively).

**Table 3:** Examination of the number of available products and product application type per review

Reviews included in dataset	Therapeutic area	Safety issue system organ class^a^	Duplicate reports	Total available products	Product type		
			*N* = 901 (%)^b^		ANDA	BLA	NDA
Review 1 (*N* = 491)	Rheumatology	Immune system	152 (31%)	1	0	1	0
Review 2 (*N* = 506)	Pain	Metabolism and nutrition	348 (69%)	50	44	0	6
Review 3 (*N* = 199)	Neurology	Congenital	143 (72%)	35	33	0	2
Review 4 (*N* = 202)	Neurology	Nervous system	26 (13%)	1	0	1	0
Review 5 (*N* = 204)	Diabetes	Metabolism and nutrition	31 (15%)	13	0	0	13
Review 6 (*N* = 194)	Hematologic malignancy	Cardiac	55 (28%)	13	10	0	3
Review 7 (*N* = 101)	Urology	Eye	0 (0%)	1	0	0	1
Review 8 (*N* = 100)	Rheumatology	Immune system	23 (23%)	1	0	0	1
Review 9 (*N* = 96)	Psychiatry	Product issues	40 (42%)	39	36	0	3
Review 10 (*N* = 100)	Nonmalignant hematology	Product issues	24 (24%)	8	7	0	1
Review 11 (*N* = 50)	Oncology	Immune system	8 (16%)	1	0	1	0
Review 12 (*N* = 54)	Anti-infective	Cardiac	51 (94%)	33	28	0	5

*ANDA* Abbreviated New Drug Application, *BLA* Biologics License Application, *NDA* New Drug Application

^a^System Organ Class represents the highest level of MedDRA terminology hierarchy and is grouped by etiology, physiological system, or purpose

^b^Duplicate report percentage is out of total reports within each review (e.g., Review 2: 348/506)

## Discussion

In this cross-sectional analysis of duplicate reports compared with non-duplicate reports submitted to FAERS, there were several notable key themes.

Our evaluation revealed significant variability within duplicate report sets, with limited concordance across key data fields essential for case identification and management. The likelihood of concordance is influenced by factors such as the number of reports in a set and the range of possible values. For instance, sex has a more restricted set of possible values compared with PTs, which are also more susceptible to variations in coding practices. Therefore, the variations in concordance across different elements and low concordance overall are unsurprising. While this study did not directly measure the impact of these discrepancies, they undoubtedly complicate the process of duplicate detection. For signal identification analyses (e.g., disproportionality measures), removing duplicates—even with imperfect methods—is likely better than not attempting to remove them at all [16–18]. The improvement in concordance we observed following the cleaning of age and sex data suggests that additional preprocessing steps could enhance the effectiveness of matching methods for duplicate identification. Previous research demonstrated that relatively simple algorithms could augment some structured fields with high accuracy, which add feasibility to this approach since manual correction is not possible [18, 19]. But the implications of variability among duplicate sets extend beyond the performance of duplicate detection algorithms. After a duplicate set is identified, which report should be kept for calculating various measures? The first report received, the report with the highest quality metric, or maybe a new report that combines information across the set. We are not aware of any published research that has evaluated this choice, perhaps because the variability across duplicates has not been fully appreciated.

In the context of signal evaluation, there is little tolerance for duplicate misclassification and the omission of important case details. Assessors manually verify duplicates and consolidate data across reports into one “case” that represents the most complete facts when creating a case series. Importantly, we didn’t assess if these differences between narratives in duplicate sets were meaningful; however, based on our experience, there are instances where critical information appears in one report but is absent in another within a set of duplicates. As anticipated, narratives among duplicate sets exhibited greater similarity compared with non-duplicates across all reviews, as indicated by higher cosine similarity scores. To explore if this finding remained consistent when comparing narrative similarity between duplicates pairs and non-duplicates pairs within a single PV review—where narratives may be more similar regardless of duplication because the reports likely involve the same products/adverse events of interest—we ran a post hoc analysis examining within-review pairs. We used a cutoff of cosine similarity score of 0.76 (sensitivity = 92% and specificity = 70%), which resulted in cosine similarity scores of 0.87 (duplicates) and 0.67 (non-duplicates), showing the difference between groups remained but was less appreciable.

On the basis of our primary and post hoc analyses findings, the cosine similarity measure appears to be a promising tool for facilitating duplicate identification. However, several practical considerations remain, including computing demands (e.g., pairwise comparisons across FAERS are currently impractical given the large volume of reports received daily), model and threshold selection, the potential need for context-specific configurations, and integration in human-in-the loop workflows [16, 20, 21]. In addition, combining cosine similarity with other approaches could enhance accuracy and robustness.

We found that duplicate submissions in our dataset were often derived from other regulatory authorities’ databases and more likely to be foreign reports. This is likely an unintended consequence of reporting regulations and the increased accessibility of PV databases such the Canada Vigilance adverse reaction online database [22]. In 2018, the EMA launched a pilot that requires certain marketing authorization holders (MAH) to monitor EMA’s EudraVigilance database and inform EMA and national competent authorities of validated signals with their medicines [23]. During MAH’s monitoring, they may gain awareness of reports submitted by other MAHs—and consequently may be required to submit them to other authorities such as the FDA (who may already have the reports from the first MAH). Although reporting requirements may lead to duplication, the intent is to prevent missing information that could hinder effective signal management. Others have recently highlighted inefficiencies and other potential problems caused by replication of adverse event reports between databases [24]. The medical literature is another well-known source of duplicates that was also evident in our dataset [4, 5]. Proposed revisions to ICH E2D emphasize that the literature reference should be adequately recorded in the report using the recommended format for citations in ICH E2B (i.e., Vancouver style) to facilitate duplicate report management [3]. The generalizability of this study’s findings to other adverse event reporting systems may be limited owing to special programs (e.g., EMA’s literature monitoring services [25]) and differences in local and regional reporting requirements.

Our study has several strengths. First, although FAERS data is publicly available, it does not include case narratives. Due to the unique ability to examine unredacted case narratives and structured data elements, we were able to characterize duplicate reports with greater precision adding to the robustness of our findings. This was particularly important for our characterization of report sources, which has not been well documented in literature. Second, we used a large dataset of reports across multiple drug events and therapeutic areas across time to assess duplicate report characteristics. Third, we explored a novel NLP method to examine the feasibility of using case narratives to examine and detect overlaps in unstructured texts between duplicate reports. Conversely, we recognize there are several limitations of our findings. Our dataset contained a non-random convenience sample of reports which may impact the generalizability of our findings. Nonetheless, the sample may be considered enriched, as focused reviews likely contain a higher proportion of duplicates compared with the FAERS database overall. Across the included reviews, we observed a wide range in the proportion of duplicates (0–94%), consistent with prior analyses [7]. The classification of reports into source categories reliant on simple text searches of the narrative and manual evaluation of reports was not based on a validated tool. Lastly, we utilized randomly selected non-duplicate reports from the entire dataset as a reference measure for the SBERT narrative similarity analysis; this approach may have resulted in a greater distance observed between non-duplicate and duplicate groups than if we had limited the non-duplicate report pairing to those with the same drug–event focus.

In the absence of unique global report identification numbers, the detection and handling of duplicate reports will remain an essential element of good case management. Advances in tools that leverage developments in artificial intelligence and efforts to harmonize regulatory requirements, where feasible, will be key to addressing this universal challenge effectively.

## Conclusions

We observed differences in the attributes of and potential contributors to duplicate reports in FAERS that may inform duplicate prevention, detection, and management strategies. However, further studies are needed to better understand the implications of these findings and how potential regulatory changes and technological advances can be leveraged to further address duplicate reporting in adverse event reporting systems.

## Supplementary Information

Below is the link to the electronic supplementary material.

Supplementary file1 (PDF 168 KB)

## Declarations

### Data availability

The datasets used in the current study contain information that cannot be disclosed such as personal identifiers. Although data archiving is not mandated, select data that is able to be disclosed will be made available from the corresponding author on reasonable request.

### Code availability

The analytic code can be made available upon request to the corresponding author.

### Disclaimer

This article reflects the views of the authors and should not be construed to represent the FDA’s views or policies.
