# Correction: An Evaluation of Duplicate Adverse Event Reports Characteristics in the Food and Drug Administration Adverse Event Reporting System

**DOI:** 10.1007/s40264-025-01585-y

**Published:** 2025-07-25

**Authors:** Scott Janiczak, Sarah Tanveer, Karen Tom, Rongmei Zhang, Yong Ma, Lisa Wolf, Monica A. Muñoz

**Affiliations:** 1 Office of Surveillance and Epidemiology (OSE), Center for Drug Evaluation and Research (CDER), US Food and Drug Administration, Silver Spring, MD USA; 2 Office of Strategic Partnerships and Technology Innovation, Center for Devices and Radiological Health, US Food and Drug Administration, Silver Spring, MD USA; 3 Office of Biostatistics, US Food and Drug Administration, Silver Spring, MD USA

**Correction: Drug Safety** 10.1007/s40264-025-01560-7

In this article, the authors identified and corrected errors in part of the dataset, which affected the results presented in the original publication. The errors stemmed from the inadvertent provision of an incorrect dataset to the authors, which was originally intended for unrelated research. Because of the changes in the proportion of duplicate reports/sets and non-duplicate reports within the dataset, numerical data throughout the Results section were updated. These revisions did not materially alter the overall findings, and no significant alterations were required in the Key Points, Discussion, or Conclusions sections of the original publication.

The original article has been corrected.

