# Supplementary material for: An Evaluation of Duplicate Adverse Event Reports Characteristics in the Food and Drug Administration Adverse Event Reporting System
Source: Drug Saf. 2025 Jun 4;48(10):1119–26. doi: 10.1007/s40264-025-01560-7 (PMC12423208; doi:10.1007/s40264-025-01560-7)
Supplement: Supplementary file 1 — Supplementary file1 (PDF 168 KB) [file 40264_2025_1560_MOESM1_ESM.pdf]

Electronic Supplemental Material (ESM)

TITLE

An evaluation of duplicate adverse event reports characteristics in the Food and Drug Administration Adverse Event Reporting System

AUTHOR INFORMATION

Authors: Scott Janiczak, PharmD, MPH[1], Sarah Tanveer, MA[1], PhD, Karen Tom, PharmD[2], Rongmei Zhang, PhD[3], Yong Ma, PhD[3], Lisa Wolf, PharmD[1], Monica A. Muñoz, PharmD, PhD[1]

Affiliations:

1. Office of Surveillance and Epidemiology, Center for Drug Evaluation and Research, US Food and Drug Administration, Silver Spring, MD USA
2. Office of Strategic Partnerships and Technology Innovation, Center for Devices and Radiological Health, US Food and Drug Administration, Silver Spring, MD USA
3. Office of Biostatistics, US Food and Drug Administration, Silver Spring, MD, USA

Disclaimer: This article reflects the views of the authors and should not be construed to represent FDA's views or policies.

CORRESPONDING AUTHOR

Scott Janiczak, PharmD, MPH, BCPS
Office of Surveillance and Epidemiology (OSE)
Center of Drug Evaluation and Research (CDER)
US Food and Drug Administration
Scott.Janiczak@fda.hhs.gov

eTable 1. Example duplicate sets with cosine similarity scores near the median (0.76) for duplicate pairs

Example Set/Report (cosine similarity score)	Report Narrative
Set 1/Report 1 (0.93)	This is a regulatory report from the UNITED KINGDOM OF GREAT BRITAIN AND NORTHERN IRELAND of CONGENITAL HAND MALFORMATION, FOETAL EXPOSURE DURING PREGNANCY and AMNIOTIC BAND SYNDROME in a neonate patient (age and gender not reported) coincident with ondansetron (for reporting purposes coded to Ondansetron 2mg/ml Solution for Injection) therapy. This report was received by the UNITED KINGDOM OF GREAT BRITAIN AND NORTHERN IRELAND Regulatory Authorities (reference number: GB-MHRA-ADR 24287718 and GB-MHRA-EYC 00174290) via a Physician and forwarded to Baxter. SUSPECT PRODUCT DETAILS: On an unreported date, the patient (neonate) was exposed in-utero to ondansetron and cyclizine (doses, frequencies and lot numbers not reported) via transplacental route [(mother's routes, doses, frequencies and lot numbers not reported) for hyperemesis gravidarum]. The action taken with respect to the event of congenital hand malformation was unknown for ondansetron and not applicable for cyclizine. The action taken with respect to the event of drug exposure in-utero was not applicable for both the suspect drugs. EVENT DETAILS: On an unreported date, the patient had DRUG EXPOSURE IN-UTERO. On an unreported date, the patient experienced CONGENITAL HAND MALFORMATION (congenital anomaly/Birth defect). Treatment rendered for the event was not reported. OUTCOME: CONGENITAL HAND MALFORMATION: Unknown and DRUG EXPOSURE IN-UTERO: Recovered/resolved. MEDICAL HISTORY: Not reported. CONCOMITANT THERAPY: Not reported. CAUSALITY ASSESSMENT: CONGENITAL HAND MALFORMATION: Primary Reporter and Health Authority: Not reported for both the suspect drugs. DRUG EXPOSURE IN-UTERO: Primary Reporter and Health Authority: Not applicable for both the suspect drugs. Baxter captured DRUG EXPOSURE IN-UTERO as an additional non-serious event. Baxter captured cyclizine as a co-suspect drug. This is one of the multiple reports received from the same reporter. FOLLOW-UP INFORMATION (30May2018): Follow-up information was received from a Physician. Adverse event information and suspect product details were added or revised. The event of DRUG EXPOSURE IN-UTERO was removed. SUSPECT PRODUCT DETAILS: On 02Sep2017, the patient was exposed to ondansetron and cyclizine via transplacental route. The action taken with respect to the event of congenital hand malformation was dose maintained for both the suspect drugs (previously unknown for ondansetron and not applicable for cyclizine). EVENT DETAILS: The last menstrual period (LMP) date of the patient mother was on 12May2017. It was reported that, the mother underwent pregnancy scan and the result was normal. The mother received cyclizine for hyperemesis gravidarum (as a past drug). FOLLOW-UP INFORMATION (18Jan2022): Follow-up information was received from a physician. Adverse event information, suspect product details and causality were added or revised. The events of FOETAL EXPOSURE DURING PREGNANCY and AMNIOTIC BAND SYNDROME were added. SUSPECT PRODUCT DETAILS: On 02Sep2017, the patient (neonate) was exposed in-utero to ondansetron and cyclizine due to hyperemesis

	gravidarum. The action taken with respect to the events of FOETAL EXPOSURE DURING PREGNANCY and AMNIOTIC BAND SYNDROME was dose maintained for both the suspect drugs. EVENT DETAILS: It was reported the patient mother had previous pregnancies without complications. On an unreported date, the patient experienced serious events of FOETAL EXPOSURE DURING PREGNANCY, AMNIOTIC BAND SYNDROME and congenital malformation (further described as absent middle and distal phalanges). Treatment rendered for the events was not reported. OUTCOME: FOETAL EXPOSURE DURING PREGNANCY: Unknown, AMNIOTIC BAND SYNDROME: Unknown. CAUSALITY ASSESSMENT: FOETAL EXPOSURE DURING PREGNANCY, AMNIOTIC BAND SYNDROME: Primary reporter and Health Authority: Not reported for both the suspect drugs. Ondansetron 2mg/ml Solution for Injection was used OFF label FOR (Off Label Use in Primary Source Country - Route).
Set 1/Report 2 (0.93)	Regulatory Authority report received on 23-Apr-2018. This case, received from a physician in the United Kingdom, involved a neonate patient with unknown age and sex who was reportedly exposed to ondansetron during gestation and experienced congenital hand malformation. Medical history of parent (mother) included hyperemesis gravidarum and previously used cyclizine. Concomitant medications were cyclizine. Unknown Date: The patient's mother initiated (transplacental, unknown frequency and route) for hyperemesis gravidarum. The child patient was exposed to ondansetron during pregnancy via transplacental route and developed congenital hand malformation. The event congenital hand malformation and foetal exposure during pregnancy were unknown. Follow-up Information was received by Mylan on 17-Oct-2018 via the United Kingdom, Regulatory Authority (Authority Reference No. GB-MHRA-EYC 00174290): Added medical history for parent. Upgraded cyclizine from concomitant medication to non-company suspect. Updated outcome of the event Foetal exposure during pregnancy. Medical history of patient’s mother includes scan (pregnancy scan normal). Non-company suspect included cyclizine. The patient was exposed to cyclizine at an unknown dose and frequency via transplacental route for hyperemesis gravidarum. The event foetal exposure during pregnancy was resolved. Follow-up information was received from the British regulatory authority, (Authority Reference No. GB-MHRA-EYC 00174290) on 18-Jan-2022 which is significant. The following information was added. Medical history, Event added, suspect start date added. Patient’s mother medical history included pregnancy. 02-Sep-2017: The patient’s mother initiated ondansetron and cyclizine at unknown dose and frequency via unknown route for hyperemesis gravidarum and patient was exposed via transplacental route. Unknown date: The patient born with absent middle and distal phalanges on right hand. Possibly amniotic band or other developmental abnormality however could not exclude teratogenicity. The event amniotic band syndrome which was medically significant. The outcome of event amniotic band syndrome was unknown. Company comment: Serious: Congenital hand malformation, amniotic band syndrome are unlisted events and foetal exposure during pregnancy (non-serious) is listed event as per company RSI of ondansetron. Causality of the reported events assessed as possible, as contributory role of suspect drug cannot be completely excluded considering the available information.

Set 2/Report 1 (0.78)	A nurse activated the enoxaparin safety needle after administering a dose to a patient. While holding the plastic part in her left hand, the yellow part attached to the needle fell out the bottom and poked her right thigh. The injection device somehow fell apart in to those two parts. She immediately let the charge nurse know who gave her an Accident Reporting and Treatment form as well as the Workman's Comp form. The nurse went to emergency department triage to be evaluated for the needle stick.
Set 2/Report 2 (0.78)	Initial information regarding an unsolicited valid non-serious case received from a other health professional in United States and transmitted to Sanofi on 14-May-2019. This case involves a patient who reported needle stick/ puncture and needle broken, while he/she was treated with Enoxaparin sodium with the use of medical device Enoxaparin pre-filled syringe. The patient's past medical history, medical treatment, vaccination and family history were not provided. On an unknown date, the patient was started on enoxaparin sodium (Formulation: unknown; Dosing details: Unknown) (Batch number: 8L938A; Expiration date: 30-Sep-2021) with the use of medical device Enoxaparin pre-filled syringe. It was reported that, a nurse activated the enoxaparin safety needle after administering a dose to a patient. While holding the plastic part in her left hand, the yellow part attached to the needle fell out the bottom and poked her right thigh. The injection device somehow fell apart in to those two parts. She immediately let the charge nurse know who gave her an Accident Reporting and Treatment form as well as the Workman's Comp form. Action taken and corrective treatment were not applicable. The event outcome was unknown for injury associated with device and not applicable for needle issue. No further relevant information was reported. Follow-up information regarding an unsolicited valid non-serious case received from a other health professional in United States and transmitted to Sanofi on 22-May-2019. Report contains no new information. Additional information was received for enoxaparin sodium (Batch number: 8L938A; Expiration date: 30-Sep-2021) form PTC with reference PTC number: 1001014432 on 04-Jun-2019. Sample status: Not available. Company language: case summary Based on the investigation performed, the batch was manufactured according to our SOP During the ICC syringes with scratches or with impact were noticed but the result was within the defined AQL No unusual event was recorded. The retained samples met the specifications No sample was provided for evaluation Therefore, no clear root cause was raised. Please see the files attached) VHE - PTC Assistant - 04-Jun-19. Summarized conclusion: No assessment possible.

eFigure 1. Distribution of cosine similarity analysis of narrative text, stratified by review group

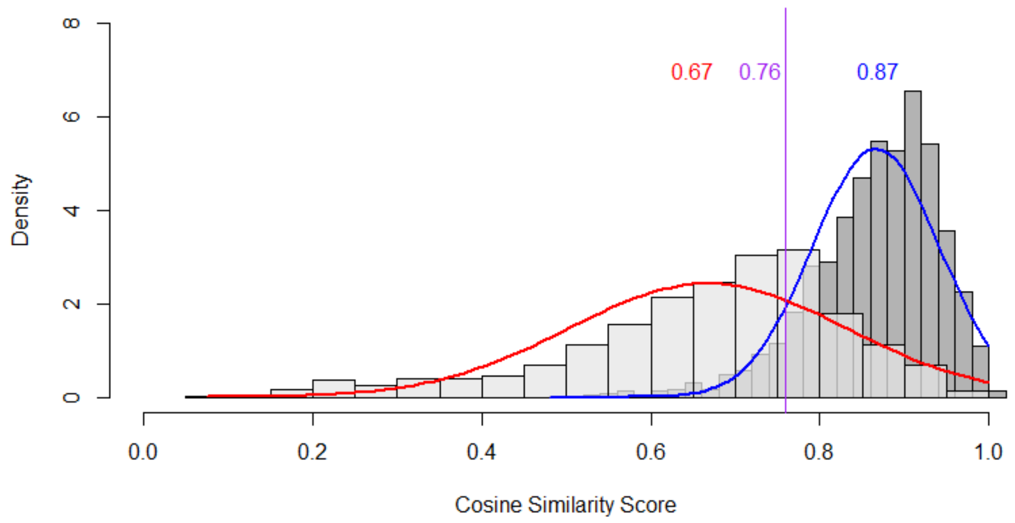

Note: When a cutoff of cosine-similarity score of 0.76 was used, sensitivity is 92% and specificity is 70%. The red-line represents non-duplicate pairs, and the blue-line represents duplicate pairs.
